# Pharmacotherapy Evolution in Alzheimer’s Disease: Current Framework and Relevant Directions

**DOI:** 10.3390/cells12010131

**Published:** 2022-12-28

**Authors:** Denisa Claudia Miculas, Paul Andrei Negru, Simona Gabriela Bungau, Tapan Behl, Syed Shams ul Hassan, Delia Mirela Tit

**Affiliations:** 1 Doctoral School of Biomedical Sciences, Faculty of Medicine and Pharmacy, University of Oradea, 410087 Oradea, Romania; 2 Department of Pharmacy, Faculty of Medicine and Pharmacy, University of Oradea, 410028 Oradea, Romania; 3 Department of Pharmacology, School of Health Sciences & Technology (SoHST), University of Petroleum and Energy Studies, Bidholi 248007, India; 4 Shanghai Key Laboratory for Molecular Engineering of Chiral Drugs, School of Pharmacy, Shanghai Jiao Tong University, Shanghai 200240, China

**Keywords:** Alzheimer’s disease, cholinesterase inhibitors, glutamate antagonists, cognitive improvement, symptom reduction

## Abstract

Alzheimer’s disease (AD), once considered a rare disease, is now the most common form of dementia in the elderly population. Current drugs (cholinesterase inhibitors and glutamate antagonists) are safe but of limited benefit to most patients, offering symptomatic relief without successful cure of the disease. Since the last several decades, there has been a great need for the development of a treatment that might cure the underlying causes of AD and thereby slow its progression in vulnerable individuals. That is why phase I, II, and III studies that act on several fronts, such as cognitive improvement, symptom reduction, and enhancing the basic biology of AD, are imperative to stop the disease. This review discusses current treatment strategies, summarizing the clinical features and pharmacological properties, along with molecular docking analyses of the existing medications.

## 1. Introduction

Alzheimer’s disease (AD) is a degenerative and irreversible brain condition that affects memory, cognition, and eventually the ability to perform even the most basic tasks. After detecting anomalies in the brain of a patient who died of an undiagnosed mental disorder (eventually identified as amyloid plaques and tau fiber bundles), Dr. Alois Alzheimer named the disease after him [1]. 

Damage appears to occur initially in the hippocampus and entorhinal cortex, parts of the brain that are essential in forming memories [2]. Other brain areas are damaged as more neurons die, and brain tissue is drastically diminished in the final stages of AD [3]. While many factors, such as genetics and lifestyle, influence a person’s chance of developing AD, age is by far the most significant; the disease is rare before the age of 65, and the recurrence rises in later decades, with a 24–33% chance of developing the disease by the age of 85 [4]. Much has been learned about the biological foundation of the illness in the previous three decades, emphasizing the potential for generating biomarkers for diagnosis, risk assessment, clinical trials, therapeutic targeting, and identifying novel pharmacological targets. Despite these advancements, only a few medications for AD have been licensed, and these are symptomatic therapies that do not stop progressive neurodegeneration; instead, they change the disease’s trajectory by stabilizing or delaying it and hence are unsuccessful in treating it [5]. The failure to develop drugs capable of treating the causes of this disease is due to insufficient knowledge of the mechanisms that characterize this disease [6].

Molecular docking can provide useful information on the design of new inhibitors, reducing development time and costs, improving the efficacy of the study substance, and minimizing the time and costs of chemical synthesis and biological testing. It also allows the simulation of the mechanisms of action of drug substances and the prediction of therapeutic dose values, as well as the optimization of pharmacokinetic properties, the discovery and validation of new targets, and the reduction of adverse drug effects [7].

This review discusses current treatment strategies, summarizing the clinical features and pharmacological properties, along with personal molecular docking analyses (of the authors of this paper) related to the available medications for the management of cognitive impairment and dysfunction in global activities in symptomatic AD. The modern molecular docking approaches were performed using AutoDock Vina 1.5.4 [8], and the data were interpreted using Discovery Studio [9] and UCSF Chimera [10].

The objective of this review is to compile and assess the most recent information on prospective AD disease-modifying medicines that have been created or are under investigation. Of particular focus are therapies that are being assessed through clinical trials. With an impartial and methodical presentation of the data, this review helps readers understand the progress made thus far and the potential for therapeutic approaches in the near future. Additionally, the molecular docking analysis offers a deeper understanding of the mechanism of action of current drugs used in this field and underlines the increased potential to significantly improve drug discovery, drug repositioning, and virtual screening of chemical libraries. By committing to a comprehensive and collaborative approach to understanding the disease and finding new, effective, and safe treatment options, we can overcome the challenges currently faced in treating AD and bring about positive change in the near future.

## 2. Treatment of Alzheimer’s Disease

AD is a complicated disease that is unlikely to be successfully treated by a single medicine or other intervention [11]. Current pharmacotherapeutic techniques are centered on assisting patients in preserving mental capacities, managing behavioral manifestations, and delaying the progression, thereby slowing illness symptoms’ emergence. All currently available therapies function by modulating the amounts of certain neurotransmitters in the brain, primarily acetylcholine (ACh) and glutamate.

### 2.1. Acetylcholine and Acetylcholinesterase

Alzheimer’s disease has been linked to ACh deficiency in the brain because a decrease in ACh levels leads to impaired cognitive function [12]. As a result, the hypothesis is that boosting ACh availability by blocking acetylcholinesterase (AChE, a cholinergic enzyme that hydrolyses ACh) might reduce the evolution of cognitive decline. Inhibiting the breakdown of ACh by blocking AChE has been found to help reduce the advancement of the disease, even though there is no effective cure [13]. 

**Figure 1:**
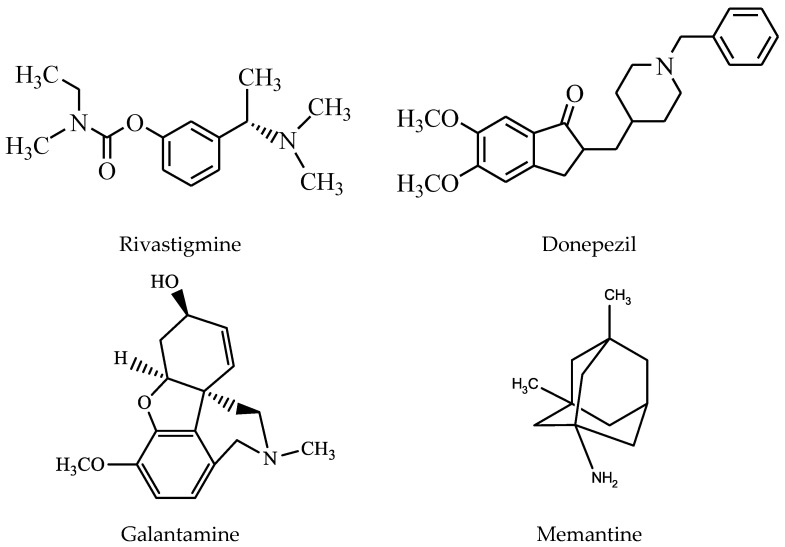
The main drugs and their chemical structure approved in the treatment of Alzheimer’s disease.

Currently, donepezil, rivastigmine, and galantamine (Figure 1) are the only AChE inhibitors approved by the Food and Drug Administration (FDA) for the treatment of AD. They act by reversibly binding and inhibiting AChE, increasing acetylcholine levels [12]. The first AChE inhibitor approved by the FDA, tacrine, was discontinued because of the numerous side effects. Besides the central nervous system, ACh is also found in the parasympathetic vegetative nervous system [14], slows the heart rate, and stimulates the gastrointestinal tract and bladder. Predominantly present at the periphery, butyrylcholinesterase (BuChE), also hydrolyses ACh [15]. 

### 2.2. AChE Enzyme Sites

AChE has an active site represented by a catalytic triad (commonly found in hydrolase enzymes), which is responsible for the hydrolysis of the ester bond. The residues that form the catalytic triad are Ser203, His447, and Glu334 [16]. 

The active site of acetylcholinesterase interacts with acetylcholine at two subsites, the catalytic anionic site (CAS) and the peripheral anionic site (PAS) [17]. At the entrance of the protein’s aromatic pocket, we can find the peripheral anionic site, which, by interacting with the beta-amyloid peptide, leads to a faster aggregation of amyloid plaques. The catalytic anionic site is responsible for the correct orientation and stabilization of the trimethylammonium group of acetylcholine.

AChE inhibitors used in AD should target PAS. According to some molecular docking studies, donepezil bind to both PAS and CAS simultaneously; tacrine and galantamine bind only to CAS [18,19,20]. 

Trp86 is one of the most important aromatic residues for ACh binding since mutations of this residue result in lower reactivity. The active site’s active pocket is approximately 20 A deep. The preponderance of ligands is located near the bottom of the hydrophobic pocket, with larger ligands such as donepezil reaching all the way to the aperture [21]. The enzymatic activity of AChE is decreased due to a steric hindrance and caused by the ligand bound to PAS [22]. In the synthesis of ACh from choline, choline acetyltransferase (ChAT) acts as a catalysator and CoA as a substrate.

The reduction of the cholinergic system is one of the most noticeable biochemical processes, with decreased activity of AChE and acetylcholine transferase, as well as low levels of acetylcholine. ACh is made from choline using acetyl CoA as a substrate in a ChAT-catalyzed process. Second, presynaptic neuronal membranes fuse with synaptic vesicles that hold ACh, releasing ACh, which interacts with postsynaptic neuron receptors. ACh is then hydrolyzed by AChE, resulting in acetate and choline [13]. 

### 2.3. Response to Treatment with AChE Inhibitors

The relief of symptoms by the administration of cholinesterase inhibitors results in the return of the clock to the disease by at least 6–12 months. The screening tools used as common knowledge measures are the Mini-Mental State Examination (MMSE) and the Montreal Cognitive Assessment (MoCA) [23]. Improving the score by 2 or 3 points on MMSE and MoCA occurs when the treatment is successfully administered. 

There is variability in the response in terms of the benefits and side effects of a drug. If there is no improvement but only a stabilization or a decline and/or a significant decline in side effects after cholinesterase inhibitor treatment, another cholinesterase inhibitor may be tried [24]. 

### 2.4. Side Effects of AChE Inhibitors

Inhibiting AChE leads to the activation of the parasympathetic nervous system (PSNS), which causes the most common side effects of this class of medication: bradycardia, syncope, and gastrointestinal issues such as nausea, vomiting, and diarrhea [25,26]. For most patients, the gastrointestinal issues subside in a few days, but in some rare cases, the administration of the drug must be stopped or the dose lowered due to severe side effects. Some drugs in this class, rivastigmine and tacrine, also inhibit BuChE, but like in the case of AChE, this can lead to PSNS activation. [27]. 

Vivid dreams occurring during rapid eye movement (REM) sleep are one of the most common side effects [28]. They are described as pleasant, painful, or neutral by patients. The administration of the therapy in the morning helps to minimize the unpleasant ones. Dizziness, sleeplessness, headache, muscular cramps, rash, seizures, or heart rate slowing are some of the less usual adverse effects [29]. 

## 3. Cholinesterase Inhibitors

### 3.1. Donepezil

Donepezil is an acetylcholinesterase inhibitor, which acts selectively and reversibly, decomposing acetylcholine [30]. Most pharmacological activities of this drug are thought to happen as a result of this chemical restraint, expanding cholinergic transmission [31].

Donepezil is the second-longest-acting AChE inhibitor, having been on the shelf since 1996. Efficacy was first tested in people with mild to moderate dementia and later in people with severe dementia. As a result, it is the only inhibitor that has been licensed for use in all stages of AD. Donepezil positively affects symptoms such as hallucinations, poor concentration, and lethargy in general [32]. 

Donepezil, 1-benzyl-4-[(5,6-dimethoxy-1-indanone)-2-yl] methyl piperidine hydrochloride (E2020), is a derivative of indanone benzyl piperidine with selective, reversible AChEI activity in the CNS and other tissues [33,34,35]. Donepezil is about 10 times more potent than tacrine as an AChE inhibitor and 500–1000 times more selective for AChE than butyrylcholinesterase (BuChE). This compound is slowly absorbed from the gastrointestinal tract and has an elimination half-life of 50–70 h in young volunteers (>100 h in elderly subjects) [36]. After extensive liver metabolism, the parent compound is 93% bound to plasma proteins [37]. More recent studies indicate that donepezil is 40–500 times more potent than galantamine in inhibiting AChE. The elimination of galantamine from the brain is faster than donepezil [38].

**Figure 2:**
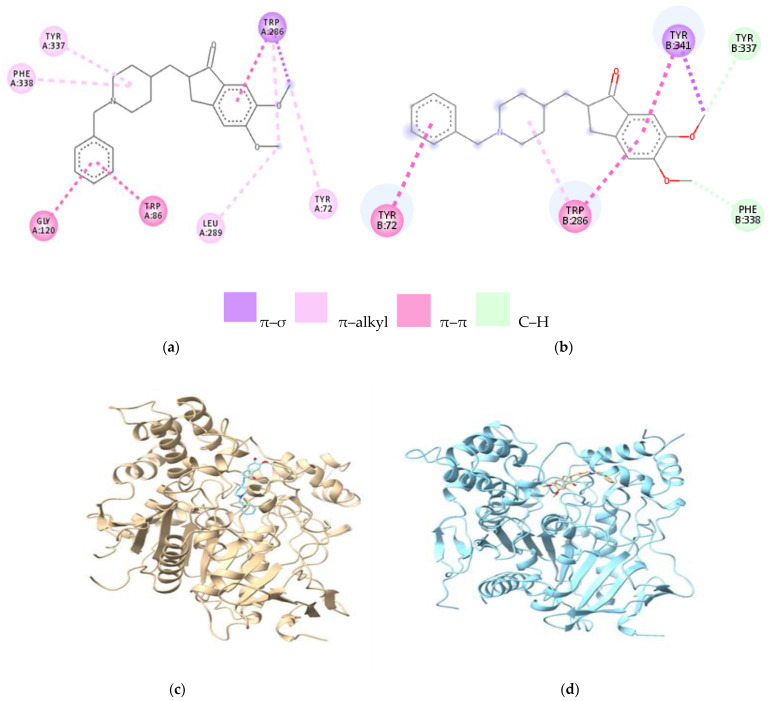
2D and 3D representation of the ligand–protein complex; (**a**) donepezil–AChE (A chain) complex; (**b**) donepezil–AChE (B chain) complex; (**c**) 3D structure of donepezil–AChE (chain A) complex; (**d**) 3D of donepezil–AChE (chain B) complex. Legend: GLY—glycine; TYR—tyrosine; TRP—tryptophan; PHE—phenylalanine; LEU—leucine. The letters A and B represent the chain; the number represents the numbering of the amino acid.

Molecular docking was performed using the crystalline structure of recombinant human acetylcholinesterase in a complex with donepezil PDB ID: 4EY7. Donepezil binds to the A chain by van der Waals, π–σ, π–π, and alkyl bonds, each of which is represented in the 2D figure (Figure 2a). Thus, donepezil binds, by π–π type bonds, to Trp86 (distance of 4.89 Å) and Gly120 (distance of 3.95 Å) via the phenyl ring of the rest of the benzyl. Additionally, by π–π bonds, in which the phenyl nucleus from the rest of the indenone participates, it binds to Trp286. Donepezil establishes alkyl bonds with Phe338 and Tyr337 via the piperidine ring and π–alkyl bonds with Trp286 and Tyr72 via the methoxy group. In the case of docking donepezil on the B chain of AChE, the affinity is lower than in the case of docking on the A chain, resulting in a binding energy of −8.7 kcal/mol (Table 1). 

In the case of chain B (Figure 2b), the π–π bonds formed by the phenyl ring of the benzyl residue with Tyr72 predominate, respectively by the phenyl ring of the indenone ring with Trp286 and Tyr341, the carbon–hydrogen bonds (C–H) of the methyl groups with Tyr337 and Phe338, and a π bond –σ with Tyr341 (Table 1). On the two chains of the enzyme, donepezil extends from the base of the hydrophobic pocket to the opening of the pocket, binding to both CAS and PAS at the A chain level, while at the B chain, it binds only to CAS, having the same binding site as ACh. The 3D structure of the donepezil–AChE A chain complex is presented in Figure 2c, and the 3D structure of donepezil–AChE B chain is presented in Figure 2d.

After administration, the drug reaches its maximum plasma concentration in 3–4 h, and the time of administration and either the presence or absence of food do not influence absorption. In vitro, it binds to plasma proteins at a high percentage of about 96%. Equilibrium is reached after multiple doses because of the chemical structure, and it easily crosses the blood–brain barrier and has a half-life of 70 h. After oral administration, it is subjected to the first hepatic passage and is eliminated at a high percentage without modification. Coadministration with CYP3a4 and CYP2D6 inhibitors decreases the speed of donepezil metabolization [31].

The administration of doses ≥ 10 mg leads to side effects, present in up to 70% of patients, such as muscle cramps, headache, dizziness, and gastrointestinal side effects such as vomiting and diarrhea [39]. Donepezil has vagotonic effects and increases the risk of bradycardia and heart block in both patients without heart problems and those with problems [40]. 

Taking donepezil in the morning can reduce nightmares caused by the stimulation of the visual cortex [41]. Another side effect is caused by increased gastric acid secretion specific to cholinesterase inhibitors, so patients at risk for ulcers should be monitored closely. Patients with asthma or other lung diseases should be closely monitored, as cholinergic activation may lead to bronchoconstriction [42].

Donepezil is available in several pharmaceutical forms, such as standard orodispersible tablets and solutions. The initial dose is 5 mg, and after 4 weeks, it can be increased to 10 mg [43]. 

### 3.2. Rivastigmine 

To determine the beneficial effects of rivastigmine, it underwent extensive preclinical study, and the results showed that rivastigmine improved memory in the short term [44]. By minimally binding to plasma proteins, the potential for interaction with other drugs is minimal, an important feature for a drug intended to be used by the elderly who usually have other diseases and use other drugs simultaneously [45]. 

It has preferential selectivity for the hippocampus and cortex, brain regions where cholinergic deficiencies are most pronounced in AD [45]. By inhibiting both AChE and BuChE that degrades acetylcholine in the human brain, the effect will be stronger, and the benefits of treatment greater, with higher synaptic neurotransmitter levels and improved cholinergic receptor function [46]. 

With rivastigmine patches, tolerability is higher, blood levels are constant, and release is gradual over more than 24 h. The transdermal administration (TDS) system allows patients to tolerate higher/more effective doses of rivastigmine over the oral preparation [47,48,49]. By applying the transdermal patch once a day, there are benefits such as ease of administration, improved treatment adherence, and a reduction in the number of tablets in polymedicine in patients with comorbidities [50]. 

Rivastigmine is a carbamate derivative structurally related to physostigmine. It binds reversibly and inactivates AChE, preventing the hydrolysis of ACh and thus leading to an increased concentration of ACh in cholinergic synapses [51]. 

**Figure 3:**
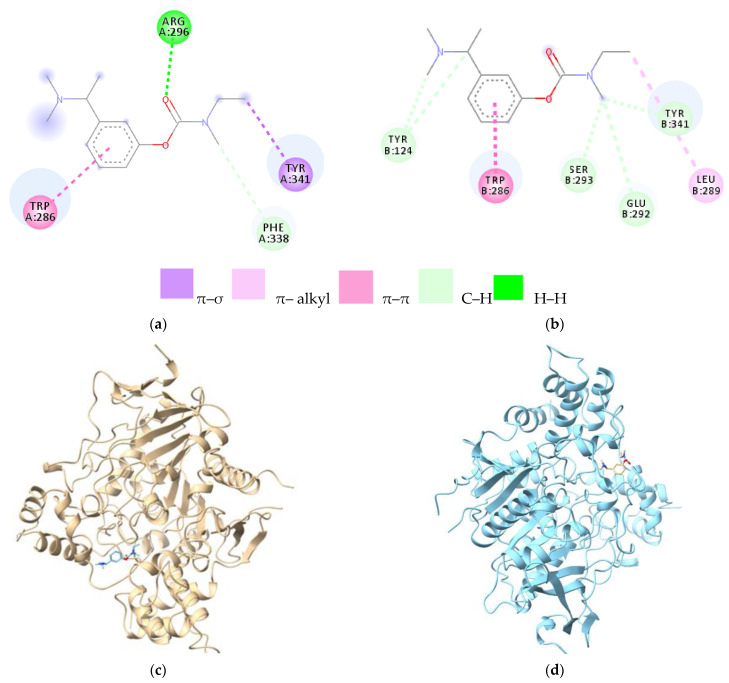
2D and 3D diagrams representing the links between ligand and protein; (**a**) rivastigmine–AChE (chain A) complex; (**b**) rivastigmine–AChE (chain B) complex; (**c**) 3D structure of the donepezil—AChE (chain A) complex A; (**d**) 3D structure of the donepezil–AChE (chain B) complex. Legend: TYR—tyrosine; TRP—tryptophan; PHE—phenylalanine; LEU—leucine; SER—serine; ARG—arginine; GLU—glutamic acid. The letters A and B represent the chains; the number represents the numbering of the amino acid.

In the case of chain A (Figure 3a), Arg296 binds through a hydrogen bond to the oxygen atom of the carbamate group, and the amino acids Tyr341 and Phe338 bind to the same carbamate group through π–σ and carbon–hydrogen bonds, respectively. In the B chain (Figure 3b), Ser293, Glu292, and Tyr341 have a common binding site to the methyl group in the ethyl–methylcarbamate residue (C–H bonds), and Leu289 binds to the ethyl group in the same moiety. Tyr124 forms carbon–hydrogen bonds with the side chain dimethyl amino group (Table 2). 

The amino acid Trp286, in both chains, through the indole ring in its structure, binds to the benzene nucleus in rivastigmine through π–π bonds. Trp286 is responsible for binding rivastigmine to the peripheral site—PAS of AChE. The 3D structure of the donepezil–AChE (chain A) complex is presented in Figure 3c, and the 3D structure of the donepezil–AChE (chain B) complex is presented in Figure 3d.

The usual dose is 3 mg, and it is administered p.o., it has a bioavailability of 36%, and the maximum plasma concentrations are reached 1 h after administration. If administered with food, the absorption is delayed, the time to reach maximum plasma concentrations is extended by 90 min, and the half-life is extended to almost 10 h [45].

Rivastigmine showed low affinity compared with donepezil to both chains A and B (−6.6 kcal/mol). In the case of chain A, Arg296 binds through a hydrogen bond to the oxygen atom of the carbamate group, and the amino acids Tyr341 and Phe338 bind to the same carbamate group through π–σ and carbon–hydrogen bonds, respectively. In the B chain, Ser293, Glu292, and Tyr341 have a common binding site to the methyl group in the ethyl methylcarbamate residue (C–H bonds), and Leu289 binds to the ethyl group in the same moiety. Tyr124 forms carbon–hydrogen bonds with the side chain dimethylamino group. The amino acid Trp286, in both chains, through the indole ring in its structure, binds to the benzene nucleus in rivastigmine through π–π bonds. Trp286 is responsible for binding rivastigmine to the peripheral site—PAS of AChE. 

Rivastigmine therapy causes gastrointestinal side effects, such as nausea, vomiting, diarrhea, and abdominal pain. These side effects can be reduced by taking medicine in two doses at the same time as food [52]. Rivastigmine causes nausea and vomiting by directly stimulating the muscarinic receptors [53]. Rivastigmine is also available as a transdermal patch in addition to syrup and pills. Patches have fewer gastrointestinal adverse effects, but they can induce erythema, edema, or dermatitis at the application site, which can be avoided by applying patches to other parts of the body [32]. 

Some disorders, such as stomach ulcers, urinary blockage, asthma convulsions, and other lung ailments, can be made worse by cholinomimetics. Constipation, gastritis, and urine incontinence are all substantially more frequent [54]. 

Rivastigmine treatment should be started with 1.5 mg twice daily for both capsules and liquid preparations. Rivastigmine is the only treatment that is available as a transdermal patch for 24 h at doses of 4.6, 9.5, and 13.3 mg [55,56,57]. 

### 3.3. Galantamine

Galantamine has higher selectivity for AChE compared with BChE. Inhibition is reversible and competitive. In addition to acting on AChE, it also acts by allosteric modulation of nicotinic receptors [58,59,60]. By competitively binding to AChE, it leads to an increase in acetylcholine. The action on nicotinic receptors modulates the release of glutamate, serotonin, and gamma-aminobutyric acid with beneficial effects in relieving the symptoms of dementia, this being an advantage over substances that act only on cholinesterase [61,62].

**Figure 4:**
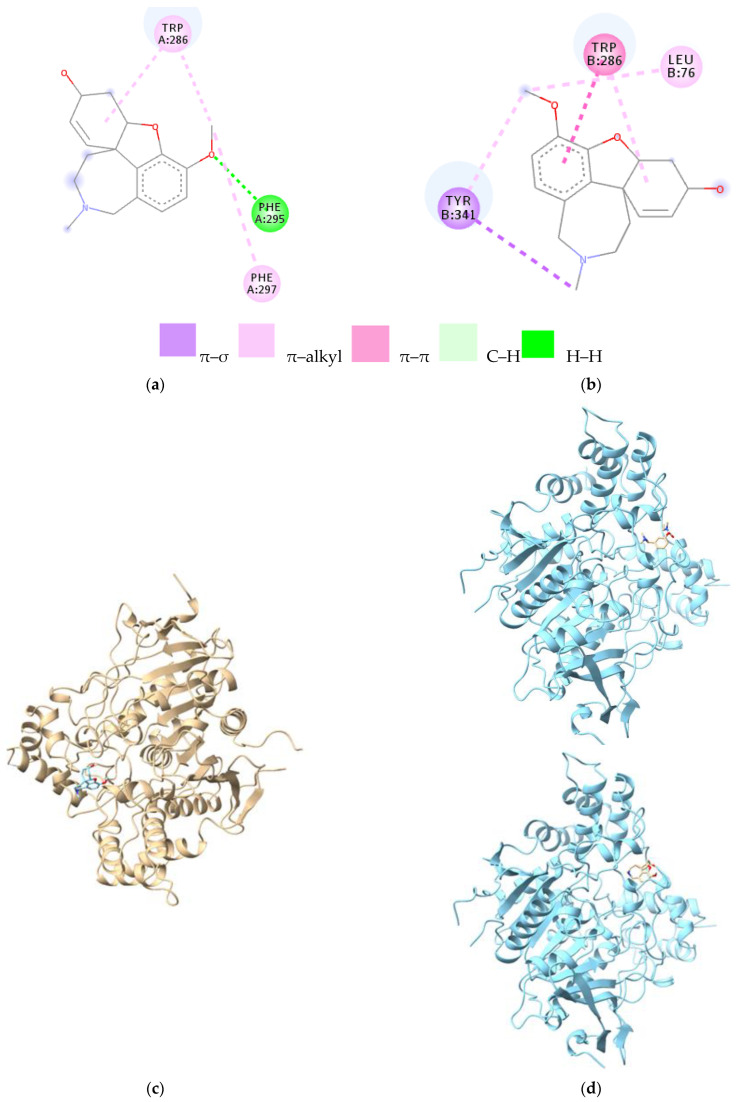
2D and 3D representation of the ligand–protein complex; (**a**) galantamine–AChE (chain A) complex; (**b**) galantamine–AChE (chain B) complex; (**c**) 3D structure of the galantamine–AChE (chain A) complex; (**d**) 3D structure of the galantamine–AChE (chain B) complex. Legend: TYR—tyrosine; TRP—tryptophan; PHE—phenylalanine; LEU—leucine. The letters A and B represent the chain, and the number represents the numbering of the amino acid.

Galantamine was the most effective medicine in reducing the symptoms of anxiety and hallucinations. It establishes π–alkyl and π–π bonds, with lengths of 4.7–4.8 Å, via cyclohexene-2-ol and benzene rings, with Trp286 in both AChE chains (Figure 4a,b). Through the oxygen atom of the methoxy group, galantamine binds to the A chain (Phe295) of the enzyme via hydrogen bonds (Table 3). 

In addition, galantamine binds through the hydrogenated azepine ring to Tyr124 (C–H bonds) and Tyr341 (π–σ bonds) to the B chain. Trp286 and Phe295 of the A chain are also found in the physostigmine–AChE complex. B chain amino acids (Trp286, Tyr124, and Tyr341) are also found in the physostigmine–AChE complex. The 3D figure of the galantamine–AChE (chain A) complex is presented in Figure 4c, and the 3D figure of the galantamine–AChE (chain B) complex is presented in Figure 4d.

It is used in dosages ranging from 8 to 32 milligrams. The bioavailability is about 100%, and maximum plasma concentrations are achieved in 1–2 h. When given with meals, absorption is slowed, and drugs that alter CYP2D6 and CYP3A4 impact the substance’s pharmacokinetics. After 3 months of treatment with 16–24 mg/day, the levels of 70 ng/mL in the cerebrospinal fluid are stable [63,64]. 

It should be taken with caution in individuals with pre-existing cardiac issues because of its mechanism of action, which might lengthen the QT interval and produce arrhythmias. The majority of these adverse effects develop at the start of therapy and gradually fade away. Galantamine had more side effects than donepezil but less than rivastigmine, according to research evaluating the adverse effects of several acetylcholinesterase inhibitors used to treat AD [64]. The daily optimal dosage varies from 16 to 24 mg, with favorable effects [65]. 

## 4. Glutamate Antagonists

### Memantine

In Europe, memantine, a glutamate antagonist used to treat AD, was approved for usage in 2003. Glutamate is a neurotransmitter that works as a partial antagonist on the N-methyl-D-aspartate glutamate receptor subtype. To obtain better outcomes in treatment, it is used with cholinesterase inhibitors [66]. 

Memantine works differently than cholinesterase inhibitors. In reality, memantine appears to have at least two therapeutically relevant modes of action: glutamate regulation and improved dopamine transmission. Glutamate transmission modulation–glutamate is the most abundant excitatory neurotransmitter in the central nervous system, with about 40% of synapses containing it [67]. Like ACh, glutamate is essential in learning and memory. Numerous preclinical studies have shown that when glutamate synapses are blocked, no new memories can form [68]. 

Glutamate crosses the synapse and affects one or more types of postsynaptic receptors when it is released from the presynaptic neuron. The N-methyl-D-aspartic receptor is one of them (NMDA). In the creation of new memories, the NMDA receptor appears to be critical [69]. 

Memantine works by regulating the NMDA receptor. Improved dopamine transmission–memantine is a dopamine agonist that stimulates dopamine receptors, increasing dopaminergic function [70]. Cognitively, memantine primarily increases attention and episodic memory (overall memory will be improved if attention is improved) [71]. Combining memantine with cholinesterase inhibitors, which function on distinct neurotransmitter systems, would have various advantages [72]. Patients normally begin therapy with a cholinesterase inhibitor and then switch to memantine after the illness has progressed to a moderate state while continuing to take the cholinesterase inhibitor [73]. In people with moderate–severe AD who do not tolerate AChE inhibitors, memantine monotherapy may be used as an alternative treatment [74]. 

**Figure 5:**
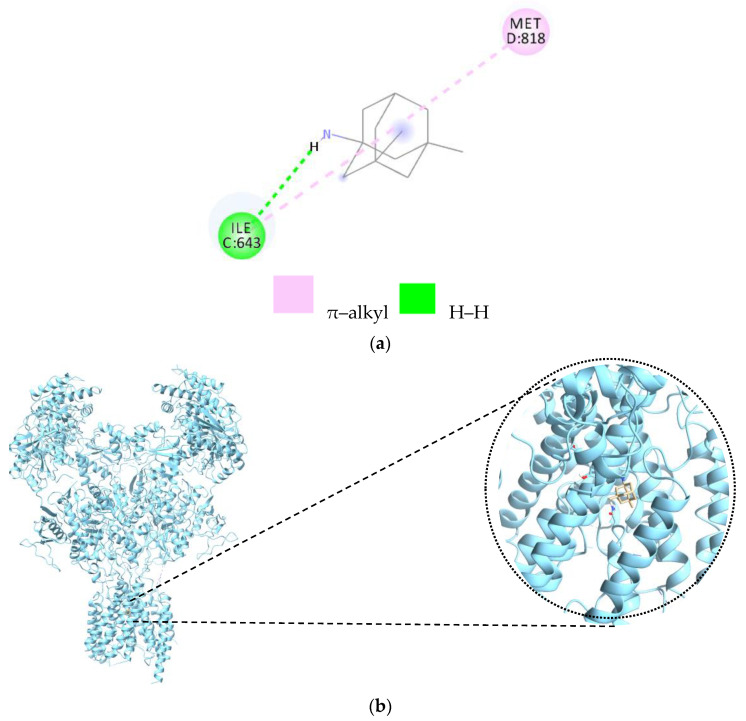
2D and 3D diagrams representing the link between memantine and NMDA; (**a**) 2D representation of the memantine- NMDA complex; (**b**) 3D representation of the Memantine—NMDA complex. Legend: ILE—isoleucine; MET—methionine; the letters C and D represent the chain, and the number represents the numbering of the amino acid.

Memantine, at the NMDA receptor, forms two bonds (Table 4). The first bond is made with the C chain of NMDA by the amino acid ILE643, and the second bond is made with the D chain by Met818, as can be seen in the 2D image (Figure 5a). The 3D complex of the memantine–NMDA complex is presented in Figure 5b.

Taking after oral ingestion, memantine is nearly totally absorbed, and food has no impact on absorption and assimilation [75]. In 3–7 h, peak drug concentrations are reached. Steady-state levels come to around day 11, with collection in plasma coming about in roughly three to four times Cmax compared with that taken after a single dosage. This drug is excreted in the urine. Roughly 48% of managed memantine is excreted unaltered in urine [76]. The most common side effects are dizziness and headache. Gastrointestinal side effects include constipation and vomiting. Confusion, hallucinations, and insomnia may also occur [77]. Memantine is given as 5 mg tablets once a day or as a solution as a starting dosage. The dose is increased from 5 mg per week to 20 mg once daily, which is recommended for at least 1 week between each dose titration [76].

## 5. Recent Progress in Medicinal Development

Because the disease acts through several mechanisms and the fact that the current treatment does not cure the disease but only alleviates the symptoms at the moment, we continue to look for drugs that can improve the lives of patients with the disease. Consequently, the trends seek for drugs that act on several fronts, such as cognitive improvement and symptom reduction, and that enhance the basic biology of BA [78]. 

Immunotherapies have garnered significant attention in recent years. Immunotherapy directs the immune system to target and destroy particular cells or substances. This type of therapy is investigated for its potential use in AD treatment. There are two types of approaches explored for treating AD: active immunotherapy and passive immunotherapy. Passive immunotherapy uses monoclonal antibodies (mAbs), proteins created to attach to and neutralize certain target molecules. A number of mAbs that target Aβ have been created and are currently undergoing clinical studies to treat Alzheimer’s disease. These mAbs are made to bind to and remove Aβ. Active immunotherapy focuses on the use of vaccines. These vaccines are made to increase the development of anti-Aβ antibodies, which may aid in removing Aβ from the brain and preventing the buildup of Aβ plaques [79]. 

### 5.1. Exploring the Molecular and Cellular Pathways in Alzheimer’s Treatment

AChE regulates impulse transmission in cholinergic pathways in the central and peripheral nervous systems. It accomplishes this by rapidly degrading the neurotransmitter acetylcholine, which is important in nerve impulse transmission. AChE, by hydrolyzing acetylcholine, aids in the termination of impulse transmission and the maintenance of proper nervous system function [80]. AChE inhibitors were created based on the fact that cholinergic pathways are disrupted in AD and other neurodegenerative diseases. Due to the fact that the cholinergic hypothesis (which served as the basis for numerous drug development approaches) does not provide a comprehensive explanation for AD’s complex pathophysiology, this theory falls short of providing a disease-modifying drug. In light of the ineffectiveness of current therapies to modify the progression of AD, significant efforts have been undertaken to identify new molecules with the potential to alter the course of the disease. 

Although the underlying molecular mechanisms of AD are complicated and still not fully understood, it is thought to involve several signaling pathways in the illness’s onset and development. The amyloid cascade hypothesis is one of the signaling pathways that has been thoroughly investigated concerning AD [81]. According to the amyloid cascade hypothesis, the buildup of amyloid-beta peptides in the brain significantly contributes to the disease [82]. The cleavage of the amyloid precursor protein (APP) by enzymes known as beta- and gamma-secretases results in the production of amyloid-beta peptides. When these peptides aggregate, they can form amyloid plaques, a hallmark of AD and a neurotoxic component of the disease [83]. According to the amyloid cascade theory, one of the main contributing factors to the onset and progression of AD is the buildup of amyloid-beta peptides. Even though many studies have suggested that Aβ aggregation plays a significant role in the development of AD, clinical trials have not consistently supported this claim, and some have even shown that amyloid-targeted therapeutics have been unsuccessful in modifying the course of symptomatic AD [84]. The reduction of amyloid plaques can be achieved mainly by reducing the production of Aβ, preventing aggregation, or by increasing Aβ clearance. Immunotherapy can achieve this by stimulating the immune system to produce its own antibodies or using exogenous antibodies. The first monoclonal antibody, approved by the FDA for the treatment of AD, is aducanumab. The main goal of aducanumab is to reduce amyloid beta, by crossing the blood–brain barrier and binding to amyloid beta proteins.

However, current research indicates that additional factors, such as the buildup of tau protein and inflammation, may also be involved in the development of the disease. The complexity of AD cannot therefore be fully explained by the amyloid cascade hypothesis [85]. As mentioned before, one of the pathways linked to AD is the tau pathway. This pathway has a crucial role in the assembly of neuronal microtubules. Its primary function as a microtubule-associated protein (MAP) is to stabilize the microtubules. Neuronal microtubules are structural components that support the form of the cell and aid in the transportation of substances inside the cell [86]. When tau is hyperphosphorylated, it can create neurofibrillary tangles that impair neurons’ ability to function normally and hasten their deterioration, leading to AD. Abnormal tau phosphorylation is a characteristic of several neurological conditions, including Alzheimer’s disease, frontotemporal dementia, and chronic traumatic encephalopathy [87]. The cyclin-dependent kinase 5 (CDK5), glycogen synthase kinase 3 (GSK3), and mitogen-activated protein kinase (MAPK) signaling pathways are just a few of the signaling pathways that control tau phosphorylation. The development of neurological diseases and tau pathology have been linked to the dysregulation of these pathways, especially the CDK5 pathway. CDK5 is most abundant in the brain and has a primary role in the development and function of neurons [88]. Tau is a protein believed to play a significant role in the progression of AD; researchers are experimenting with several methods to target tau with the goal of altering the course of the disease [84]. One approach consists of preventing tau accumulation and hyperphosphorylation. Another approach is to promote the removal of tau from the brain or to attempt to stabilize the microtubules that tau helps to maintain. Many current efforts to develop treatments for AD that target tau involve immunotherapies; this approach is still in the early stages, and no drug has reached phase III. Some researchers have suggested that using a combination of therapies may be more effective at treating AD than using a single drug. This is because of the disease’s complicated pathology and also because there may be a synergistic relationship between Aβ and tau. Therefore, utilizing drugs that simultaneously target both of these proteins may be more beneficial than a single therapy that only targets one [89,90].

Growing evidence suggests that inflammation has a role in the onset and progression of AD. Inflammation has now been observed in numerous investigations using postmortem tissues from samples of AD patients, but the role of inflammation in AD is not yet fully understood [91,92]. A change in the balance of anti-inflammatory and proinflammatory signaling, as found in AD, leads to chronic inflammation, which can lead to the activation of microglia (immune cells found in the CNS), which can produce cytokines, chemokines, and reactive oxygen species (ROS). These molecules cause damage to neurons, leading to the development of cognitive symptoms [93]. Neuroinflammation strongly contributes to AD development, which is generated by numerous damaging signals, such infection, tau oligomers, amyloid peptides, and oxidative reagents. Neuroinflammation is linked to the unusual production of proinflammatory cytokines, which activate signaling pathways, exacerbating the AD symptoms [94]. 

Overall, the complex interplay between these signaling pathways likely plays a role in the development and progression of AD, and further research is needed to understand how these pathways interact and contribute to the disease process. The complicated pathological character of AD has hampered the identification and validation of useful biomarkers for advancing its diagnostic and therapy techniques. There has been a substantial research effort to construct multi-target-directed ligands (MTDLs) for the treatment of AD, a strategy based on the understanding that AD is a composite and multidimensional illness related to numerous independent but interwoven biological pathways [95]. The main focus of MTDLs is to target multiple pathways involved in the onset of the disease. Because AD is a multidimensional illness, the approach of MTDLs could prove beneficial. Drugs targeting all or multiple pathways involved in the clearance of Aβ may prove more efficient. Another potential target for these drugs could be the pathways involved in AD-related inflammation [96,97]. 

It is anticipated that future MTDLs may offer improved efficacy against acetylcholinesterase and amyloid plaque development. Other mechanisms by which these drugs may act involve multiple pathways that lead to AD. Some drugs may have metal-complexing properties to re-establish metal homeostasis and prevent the formation of Aβ plaques. Others might act in a similar way to memantine by acting on glutamate. Blocking calcium channels, some cannabinoids, histaminic antagonists, or blocking beta-site amyloid precursor protein cleaving enzyme 1 may also prove helpful in treating AD. As a result, AD symptoms might gradually reduce, improving therapy results and patient adherence [98]. The development of novel, disease-modifying drugs will ultimately depend on the continued extensive research in this field. The significant unmet medical needs in the treatment of Alzheimer’s disease will be addressed in large part by these developments.

### 5.2. Phase I Studies

The main objective of a phase 0 study is to determine whether a certain mechanism of action determined in nonclinical models can be fulfilled in humans. In this phase, drugs that do not meet certain requirements are eliminated to move on to the study of phase I requirements, thus saving time and money [99]. 

After the successful completion of a phase 0 study, clinical trials may begin with phase I. Researchers evaluate the safety of the treatment and identify side effects, and those who participate in these studies are either healthy volunteers or patients [99]. There are 24 agents under study in phase I, 23 of which are classified as a class of disease-modifying drugs. There are 17 small molecules and 6 biological substances that are evaluated in phase I. Each study involves an average of 43 people, lasting 127 weeks (recruitment and treatment period) [100]. 

Dexmedetomidine (Precedex^®^), initially approved by the FDA in 1999, is a selective agonist of the alpha-2 adrenergic receptors. Originally used to sedate intubated and mechanically ventilated patients in intensive care, it is administered sublingually in agitation associated with dementia [101,102]. 

Emtricitabine is part of the class of nucleoside reverse transcriptase inhibitors prescribed as HIV therapy. The drug reduces a type of age-related cellular inflammation, knowing that the brain of those with AD is inflamed [103]. Trehalose improves cognition, reduces amyloid-beta deposition in the hippocampus, increases autophagy markers, and reduces neuronal death in the brain [100,104]. In the case of the two-substance study, namely, MK-1942/donepezil, the objectives include the determination of whether, together, they increase the incidence or severity of adverse events previously reported for these substances [103]. 

Trehalose, a nonreducing disaccharide, acts as the mechanistic target of rapamycin kinase complex 1 (mTORC1)–independent autophagic inductor. Autophagy induction is achieved with the help of lysosomes, protecting neurons by cleaning protein aggregates [105,106]. 

### 5.3. Phase II Studies 

During this stage, the drug is evaluated for its efficacy and the benefit–risk profile at the therapeutic dose. At this stage, it is also administered to a more significant number of people [99]. In Phase II, for the 2021 study, there are 74 agents in 87 studies, of which 30 are repurposed. Among the candidate treatments for phase II, 64 are in the class of disease-modifying therapy, 6 cognitive enhancers, and 4 drugs for behavioral symptoms. Of these, 4 of the small molecules and 7 of phase II biological substances have amyloid reduction as one of the mechanisms. Ongoing studies include all categories of patients with preclinical, prodromal, or prodromal/mild/severe and severe BA. Phase II studies include an average of 100 participants for each study category, with a mean study duration of 100 weeks (52–145 weeks) [100].

The first Aβ vaccine, AN-1792 (full-length Aβ 1–42) was tested in an active immunization clinical trial. However, because some participants developed meningoencephalitis, it was discontinued because of cytotoxic T cells or the autoimmune response [107,108]. Another vaccine is being developed, namely, ABvac40. Aβ peptides are generated from the sequential cleavage of the amyloid precursor protein (APP), as well as Aβ40 and Aβ42. Among the forms secreted by Aβ, Aβ40 is the predominant variant (90%). In the case of the Aβ42 variant, the hydrophobic oligomers are considered to be the most neurotoxic species, prone to aggregation. Prevention of the formation of toxic aggregates produced by Aβ40 is achieved by anti-Aβ40 antibodies. ABvac40 is the first active vaccine to target the C-terminus of the Aβ40 peptide [108].

Amyloid precursor protein (APP) undergoes sequential cleavages by β-secretase and γ-secretase and gives rise to β-amyloid (Aβ), responsible for dementia [109]. 

**Figure 6:**
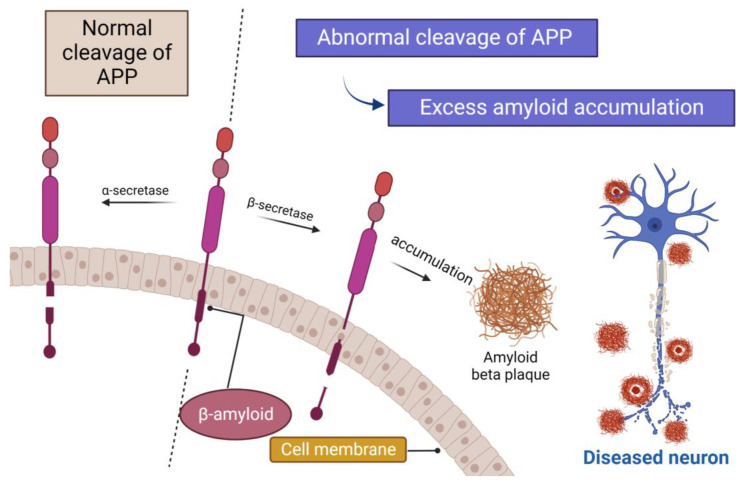
Cleavage of APP. In the normal conditions, APP is cleaved by α-secretase. In abnormal conditions, APP will be cleaved by β-secretase, resulting in the accumulation and aggregations of small peptides called β-amyloid (Aβ), with the formation of amyloid plaques.

In terms of reducing Aβ production, the three crucial enzymes that process APP have been therapeutic targets in drug development. The goal is to inhibit β-/γ-secretase while promoting α-secretase activity (Figure 6) [110]. 

In phase II studies is the enzyme α-secretase, which modulates the reduction of Aβ production (called APH-1105) acting as a DMT. AL002 is an antibody that binds to the microglial receptor, called a triggering receptor expressed on myeloid cells 2 (TREM2). Decreased TREM2 efficacy can lead to AD and other forms of dementia. Increasing the effectiveness of TREM2 can be achieved with the help of AL002, improving the survival rate and activity of microglia [100,111]. 

ACI-35 is a liposomal vaccine. It consists of a synthetic peptide antigen, corresponding to tau human protein sequence from 393 to 408 (a molecule capable of inducing an immune response) autoimmune responses of B cells or T cells against physiological forms [112,113]. In a preclinical study performed on a mouse with tauopathy, the efficacy of ACI-35 was tested, with the conclusion that long-term vaccination is safe, resulting in a reduction in tauopathy. The 6-month clinical trial 1b looked at low, medium, and high doses of ACI-35 and placebo in 24 people with mild to moderate AD. After administration of the initial doses, the booster followed, with the patients being observed for the next 6 months. At the end of the study, the conclusion was that ACI-35 triggers a weak immune response [114]. 

Simufilam re-establish the normal shape and function of the modified protein of filamin A (FLNA) in the brain. Neurodegeneration and neuroinflammation are caused by altered FLNA in the brain, which affects the normal function of neurons [115]. The filamin A protein has multiple functions and is referred to as a “scaffold protein”, which promotes the communication of brain cells; when the proteins do not function properly, AD develops [116]. 

PU-AD is an oral permeable-to-the-brain inhibitor of the heat shock protein 90 molecular chaperone (HSP90). A tau protein that has been altered or hyperphosphorylated is degraded more quickly, thanks to HSP90. Small chemical PU-AD has no effect on healthy cells, with high affinity for cancer cells and AD tissue, and has only slightly adverse effects [117,118].

### 5.4. Phase III Studies 

It is the final confirmation of safety and efficacy, which are largely controlled studies involving enough patients to have at least an 80% chance of success. The main factors to be evaluated are the effectiveness, monitoring of side effects, and comparing the medicine with the alternative therapies used regularly. The new drug is being administered to larger groups of people [99]. 

A total of 25,373 participants were required for recruitment in phase III investigations, with an average of 619 participants per study. A total of 684 people took part in prevention studies, which lasted an average of 335 weeks [100]. 

Immunotherapy targeted at preventing A aggregation has been discovered, with two mechanisms: active immunization and passive vaccination. The goal of active immunization is to develop an A42 vaccine that targets the formation of amyloid plaques. Monoclonal antibodies and immunoglobulins (Ig) are used in passive immunization. Aducanumab, gantenerumab, solanezumab, and lecanemab are some of the compounds in phase III clinical studies [119,120]. 

Aducanumab (Aduhelm^®^) was approved in 2021 following an accelerated approval process. It is the first disease-modifying pharmaceutical to be licensed, and it is well known since previous drugs approved simply relieve symptoms. It is a completely human IgG1 monoclonal antibody that binds to aggregate Aβ fibrils and decreases Aβ plaques in the brains of patients in a dose- and time-dependent manner. Clinical trials for Aduhelm were the first to reveal that reducing these plaques—a characteristic finding in the brains of Alzheimer’s patients—is predicted to slow the progression of this kind of dementia. Postapproval studies (phase IV confirmatory studies) will be conducted by drug companies to confirm the expected clinical benefit [120,121,122]. 

Coffee is a drink that has psychostimulating properties on the central nervous system due to caffeine. The pharmacological properties of caffeine allow its use as a symptomatic treatment of AD. Due to its multiple benefits, it is one of the most common protection factors. The main objective of the study is to evaluate the effectiveness of caffeine on cognitive decline in AD in the early to moderate stages, with an MMSE score between 16 and 24 [123].

Extracellular signal-regulated kinase (ERK) and activated B-cell kappa-light amplifier nuclear factor (NF-B) are two main inflammatory pathway regulators that are blocked by NE3107. It is anti-inflammatory as well as insulin sensitizing. It works by targeting numerous pathological processes in AD. It acts by bypassing the blood–brain barrier and reducing inflammatory signal transduction cascades that are known to block insulin action in the brain, restoring insulin activity [124,125]. 

A prodrug of homotaurine, ALZ801 or valiltramiprostate, is converted to 3-sulfopropanoic acid (3-SPA), which is typically found in the brain. It works by inhibiting Aβ42 aggregation and preventing the development of amyloid oligomers. In patients who carry two copies of the apolipoprotein allele 4 (APOE4/4), ALZ801 has high efficacy. The most typical side effects of ALZ801 were nausea and vomiting, which were unrelated to the dose used. These were improved by coadministration with food or 1 week after starting the medicine [126,127].

## 6. Exploring Alternative Treatments for AD

Due to the limitations of conventional treatments, the need for innovative and efficacious approaches to treat AD has grown. As a result, alternative therapies have garnered significant attention in recent years. Although alternative treatments can be highly effective in helping treat AD, some patients may decide against them due to several valid worries [128]. First, in contrast to how it controls drugs, the U.S. Food and Drug Administration (FDA) does not regulate dietary supplements. This implies that the reliability of the safety and efficacy of dietary supplements may not have been fully established [129]. Second, some supplements can impact how drugs are processed in the body, reducing their effectiveness or raising the possibility of unwanted effects [130].

Nevertheless, some dietary supplements, such as *Ginkgo biloba*, omega-3, vitamin E, and curcumin, have been investigated as possible AD treatments. However, the outcomes of these investigations have been conflicting and have not demonstrated any advantages [131]. 

Oxidative stress is the primary factor controlling ageing and several other neurological diseases. The brain’s chemical balance regulates the central nervous system’s higher functions. The human brain is especially vulnerable to oxidative stress because it consumes so much oxygen and is so high in lipids. Increased oxygen consumption generates an abundance of ROS. Polyunsaturated fatty acids, which make up the membrane of neurons, are similarly vulnerable to reactive oxygen species [132].

Antioxidants delay or mitigate cellular oxidative stress, providing several health advantages in disease prevention and therapy. They can be used alone or together with other drugs as adjuvant therapy [133]. 

The use of cannabinoids in treating AD is one of the promising treatments that is receiving an increasing amount of attention. The cannabis plant contains chemical substances called cannabinoids. Cannabinoids, notably delta-9-tetrahydrocannabinol (THC) and cannabidiol (CBD), have been linked in certain studies to the possibility of treating AD [134,135]. There is some evidence that THC and CBD may have anti-inflammatory and neuroprotective properties, as well as the potential to improve AD symptoms, including hunger and sleep. The evidence is still preliminary, and more studies are required to grasp marijuana’s therapeutic potential for AD fully [135,136,137]. It is also crucial to remember that the U.S. Food and Drug Administration (FDA) does not officially allow the use of marijuana or other cannabinoid products as a treatment for AD or any other medical condition. These drugs’ effectiveness and safety have not been extensively examined, and there could be dangers and adverse effects from using them. It is crucial to consult a healthcare professional before beginning any new treatment [135,138]. 

Because music therapy does not entail the administration of any chemicals, it is a method that any patient suffering from AD can adopt. Music is used to help people with their social, emotional, cognitive, and physical needs. It is a field that is expanding and being utilized to assist people with several illnesses, including AD [139,140]. Music therapy is not limited to listening to music, and it includes numerous activities, such as singing, playing an instrument, and producing songs. It can be tailored to fit the needs and preferences of the individual and can be performed either individually or in a group setting. It is crucial to highlight that music therapy can be used as an adjuvant therapy to help improve quality of life and address particular symptoms in people with AD rather than as a substitute for other forms of treatment, including drugs and lifestyle changes [141,142,143]. 

Other alternative treatments that might be considered for AD include aromatherapy, massage, and pet therapy. Massage therapy may assist some Alzheimer’s patients in managing their symptoms, enhancing their quality of life by reducing stress and anxiety, and improving sleep and mobility. Aromatherapy might help in stress reduction and improving sleep. Pet therapy is also known as animal-assisted therapy and could boost physical, emotional, and cognitive functions. The main benefits of animal-assisted therapy for AD patients are reducing stress, providing cognitive stimulation, and improving physical health [144,145,146]. 

## 7. Challenges

The list of unanswered questions becomes shorter as more discoveries are made in this field. Questions such as the influence of lifestyle factors, genetics, and the cause of AD still go unanswered, but with every answer, we are closer to developing a successful treatment. The major challenge of the decade in AD is certainly early diagnosis, which means creating more effective diagnostic tools. Because AD symptoms can be mild and comparable to other disorders, making a diagnosis in the early stages can be challenging [147]. 

There are currently no cures for AD, and the available therapies only temporarily alleviate symptoms and might not work for everyone. Another challenge is the potential for adverse effects of the medications used to treat AD. These medications have some adverse effects that can be unpleasant for the patient and may affect their quality of life, such as nausea, vomiting, diarrhea, and dizziness. Family and caregivers may face considerable difficulties due to the high cost of AD therapies and the stress of caring for a loved one who has the disease. The costs of drugs, in-home care, and nursing facility care can be emotionally and financially burdensome for caregivers. This is why many patients and researchers look for alternative treatments and supplements that can reduce AD symptoms. The biggest challenge in the case of plant-based supplements is the absence of strong scientific evidence to support the use of various herbal and plant-based medicines for the treatment of AD. Clinical trials have been conducted on a few herbs and plant-based medicines, but the findings have been inconsistent, and many of these trials were small or of low quality. As a result, it is challenging to determine with certainty whether these treatments are beneficial [148,149,150]. 

The main challenge for cannabinoids or cannabinoid derivates is the considerably different legal and regulatory status of cannabis and items produced from it. These products are frequently outlawed or restricted to strictly regulated medical use only. Due to this, it may be challenging for both patients and researchers to conduct controlled, rigorous studies of cannabinoid-based therapy. The use of cannabis in the treatment of AD may raise safety concerns. There is a possibility of overdose or allergic responses when using cannabis-derived drugs, some may interact with other treatments or have adverse effects. Patients should exercise caution when utilizing these treatments and speak with a healthcare provider before beginning any new course of treatment [135]. 

For those who have AD, music therapy can be a helpful intervention, but it can also present particular challenges. The use of music therapy for people with AD may present some difficulties. Due to the nature of AD, some patients may find it challenging to focus on the music for extended periods, making music therapy sessions difficult, and due to communication issues brought on by AD, patients could have problems conveying their preferences or demands during music therapy sessions. Despite these difficulties, research has demonstrated that music therapy is helpful for patients with AD. It can foster a sense of connection, communication, relaxation, and emotional well-being [141,151]. 

## 8. Conclusions

The number of people, especially the elderly, who are diagnosed with AD is growing continuously. A mix of age-related changes and genetic, environmental, and lifestyle variables is most likely responsible for the condition. Depending on the individual, these factors may have a different role in increasing/lowering the risk of AD. Aging is the most significant risk factor for AD. According to the World Health Organization, the number of persons suffering from AD will rise by at least 14% by 2025, owing to the growing population of adults over 65. Dementia has become a global problem as the world’s population ages quickly. 

Damage to cholinergic neurons in the brain and loss of neurotransmission are evident in Alzheimer’s patients. According to the cholinergic hypothesis, the main cause is the reduction of ACh synthesis. Therefore, one of the therapeutic strategies is to increase the level of acetylcholine in the brain by inhibiting the biological activity of AChE. AChE inhibitors are used to limit AChE degradation. Although they do not cure the disease, these drugs are recommended to limit neurodegeneration. The effectiveness of cholinesterase inhibitors is limited, also being the cause of side effects. That is why it is necessary to develop new therapeutic aids with different structures and mechanisms of action, studying in the same time side effects and toxicity [109]. Additionally, to increase the number of cholinesterase inhibitors available to treat the symptoms of AD, it is required to investigate a large number of substances, which translates to time and high costs. Moreover, developing a multitherapeutic drug is a difficult task. In many cases, molecular docking can achieve these requirements, which has multiple advantages: it does not require equipment or reagents, and the docking time is relatively short.

**Figure 7:**
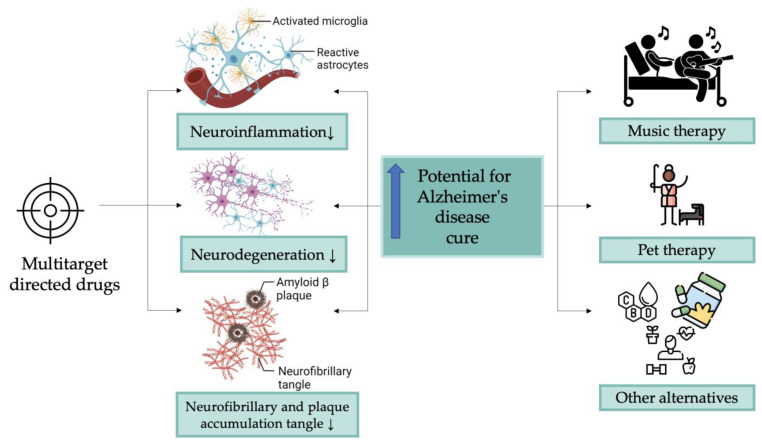
Main findings and directions of this research.

Considering all those mentioned above, the main findings of this review-type manuscript are summarized and schematized in Figure 7.

The best method to treat AD and other types of dementia is with a combination of pharmaceuticals, lifestyle modifications (such as engaging in regular exercise and eating a healthy diet), and assistance from caregivers and medical professionals. Additionally, obtaining medical aid is crucial if someone exhibits memory loss or other dementia-related symptoms because prompt diagnosis and treatment can enhance the quality of life and stop the disease’s progression. 

## Figures and Tables

**Table 1:** Donepezil–AChE ligand–receptor interactions at the A and B chains.

Amino Acids of Chain A	Distance Ligand–Protein (Å)	Types of Bonds	Amino Acids of Chain B	Distance Ligand–Protein (Å)	Types of Bonds
Gly120	3.95	π–amide	Trp286	4.97	π–alkyl
Trp86	4.92	π–π		4.46	π–π
	4.38	π–π	Tyr337	3.58	C–H
Phe338	5.02	π–alkyl	Tyr341	4.45	π–π
Tyr337	3.58	π–alkyl		3.64	π–σ
Trp286	4.34	π–π	Tyr72	4.89	π–π
	4.60	π–Alkyl	Phe338	3.40	C–H
	3.93	π–σ	-	-	-
Tyr72	5.17	π–alkyl	-	-	-
Leu289	5.15	Alkyl	-	-	-

Gly—glycine; Tyr—tyrosine; Trp—Tryptophan; Phe—phenylalanine; Leu—leucine.

**Table 2:** Rivastigmine–AChE ligand–receptor interactions at the A and B chains.

Amino Acids of Chain A	Distance Ligand–Protein (Å)	Types of Bonds	Amino Acids of Chain B	Distance Ligand–Protein (Å)	Types of Bonds
Trp286	4.87	π–π	Trp286	4.01	π–π
Arg296	2.32	leg. de H	Tyr124	3.55	C–H
Tyr341	3.62	π–σ		3.66	C–H
Phe338	3.61	C–H	Ser293	3.50	C–H
-	-	-	Glu292	3.59	C–H
-	-	-	Leu289	4.80	Alkyl
-	-	-	Tyr341	3.58	C–H

Arg, arginine; Tyr—tyrosine; Trp—tryptophan; Phe—phenylalanine; Leu—leucine; Glu—glutamic acid; Ser—serine.

**Table 3:** Galantamine–AChE ligand–receptor interactions at the A and B chains.

Amino Acids of Chain A	Distance Ligand–Protein (Å)	Types of Bonds	Amino Acids of Chain B	Distance Ligand–Protein (Å)	Types of Bonds
Trp286	4.70	π–alkyl	Tyr341	3.63	π–σ
	4.78	π–alkyl		5.14	π–alkyl
Phe295	2.87	leg. de H	Leu76	4.45	Alkyl
Phe297	4.99	π–alkyl	Trp286	4.92	π–π
-	-	-		4.08	π–π
-	-	-		4.79	Alkyl

Tyr—tyrosine; Trp—tryptophan; Phe—phenylalanine; Leu—leucine.

**Table 4:** Memantine–AChE ligand–receptor interactions at the chain.

Amino Acids	Distance Ligand–Protein (Å)	Types of Bonds
Ile643	2.86	H
Met818	4.71	Alkyl

## Data Availability

Information provided by this manuscript are supported by the References.
